# High prevalence of subclass-specific binding and neutralizing antibodies against *Clostridium difficile* toxins in adult cystic fibrosis sera: possible mode of immunoprotection against symptomatic *C. difficile* infection

**DOI:** 10.2147/CEG.S133939

**Published:** 2017-07-19

**Authors:** Tanya M Monaghan, Ola H Negm, Brendon MacKenzie, Mohamed R Hamed, Clifford C Shone, David P Humphreys, K Ravi Acharya, Mark H Wilcox

**Affiliations:** 1 Nottingham Digestive Diseases Centre, NIHR Nottingham Digestive Diseases Biomedical Research Unit, School of Medicine, University of Nottingham, Nottingham; 2 Breast Surgery Group, Division of Medical Sciences and Graduate Entry Medicine, School of Medicine, Queen’s Medical Centre, University of Nottingham, Nottingham, UK; 3 Medical Microbiology and Immunology Department, Faculty of Medicine, Mansoura University, Mansoura, Egypt; 4 Antibody Biology, UCB-New Medicines, UCB Celltech, Slough, UK; 5 Toxins Group, National Infection Service, Public Health England, Salisbury, UK; 6 Department of Biology and Biochemistry, University of Bath, Bath, UK; 7 Leeds Institute of Biomedical and Clinical Sciences, University of Leeds, Leeds, UK

**Keywords:** *Clostridium difficile*, cystic fibrosis, antibodies

## Abstract

**Objectives:**

Despite multiple risk factors and a high rate of colonization for *Clostridium difficile*, the occurrence of *C. difficile* infection in patients with cystic fibrosis is rare. The aim of this study was to compare the prevalence of binding *C. difficile* toxin-specific immunoglobulin (Ig)A, IgG and anti-toxin neutralizing antibodies in the sera of adults with cystic fibrosis, symptomatic *C. difficile* infection (without cystic fibrosis) and healthy controls.

**Methods:**

Subclass-specific IgA and IgG responses to highly purified whole *C. difficile* toxins A and B (toxinotype 0, strain VPI 10463, ribotype 087), toxin B from a *C. difficile* toxin-B-only expressing strain (CCUG 20309) and precursor form of B fragment of binary toxin, pCDTb, were determined by protein microarray. Neutralizing antibodies to *C. difficile* toxins A and B were evaluated using a Caco-2 cell-based neutralization assay.

**Results:**

Serum IgA anti-toxin A and B levels and neutralizing antibodies against toxin A were significantly higher in adult cystic fibrosis patients (n=16) compared with healthy controls (n=17) and patients with symptomatic *C. difficile* infection (n=16); *p*≤0.05. The same pattern of response prevailed for IgG, except that there was no difference in anti-toxin A IgG levels between the groups. Compared with healthy controls (toxins A and B) and patients with *C. difficile* infection (toxin A), sera from cystic fibrosis patients exhibited significantly stronger protective anti-toxin neutralizing antibody responses.

**Conclusion:**

A superior ability to generate robust humoral immunity to *C. difficile* toxins in the cystic fibrosis population is likely to confer protection against symptomatic *C. difficile* infection. This protection may be lost in the post-transplantation setting, where sera monitoring of anti-*C. difficile* toxin antibody titers may be of clinical value.

## Introduction

*Clostridium difficile* (recently reclassified as *Clostridioides difficile*)^1^ is the leading worldwide infectious cause of hospital-acquired and antibiotic-associated diarrhea. The spectrum of clinical disease varies from asymptomatic colonization to fulminant colitis and death.^2^ Current understanding indicates that *C. difficile* pathogenesis is multifactorial, dictated by pathogenic toxin production, gut microbial dysbiosis, and altered host immune and inflammatory responses.^3^

Despite heavy antimicrobial pressure and frequent hospitalization, patients with cystic fibrosis have been reported to have a high carriage of *C. difficile*, but they rarely do manifest symptoms or develop *C. difficile* infection.^4^–^10^ Asymptomatic carriers may be protected from progression to symptomatic infection because they can mount a humoral immune response to clostridial toxins.^11^ The main aim of this study was to investigate the prevalence of serum immunoglobulin (Ig)A and IgG antibodies to *C. difficile* toxins in an adult cystic fibrosis population and to determine if sera from patients with cystic fibrosis contain protective neutralizing antibodies against the toxins.

## Methods

### Study population

In this retrospective matched cohort study, all available banked sera were collected over a 3-year period beginning in 2010 from diarrhea-free adult subjects with cystic fibrosis, adult subjects with *C. difficile* infection and healthy adult controls admitted to the Nottingham University Hospitals NHS Trust and were used to investigate the ability of the microarray assay to detect the presence of IgA and IgG directed against *C. difficile* microbial toxins and control antigens. Exclusion criteria for the control group included coexisting illnesses, gastrointestinal symptoms, or treatment with antibiotics or probiotics within the previous 6 months. For the cystic fibrosis cohort, all stool and blood samples were collected within 24–48 h of commencing intravenous antibiotics. Asymptomatic carriers were defined as those without diarrhea but with a positive stool culture for *C. difficile*. All patients in the *C. difficile*–infected group had diarrhea (defined as a change in bowel habit with three or more unformed stools per day for at least 48 h) and a positive stool *C. difficile* (enzyme immunoassay) toxin test. The diagnosis of cystic fibrosis had previously been made based on a positive sweat test and/or demonstration of two known cystic fibrosis mutations and typical clinical features of the disease. Clinical and demographic information were collected from medical records. All subjects provided written informed consent for this study under the approvals granted by the Nottingham Research Ethics Committee.

### Toxin production and purification

*C. difficile* toxins A and B were purified essentially as described previously^12^ with some modifications. Starter cultures (5 mL) of the *C. difficile* strain were used to inoculate each of the 8×2 L vessels containing dialysis sacs (60 mL volume) and these were grown under anaerobic conditions for 90–96 h at 37°C. The contents of the dialysis sacs were then pooled, centrifuged at 10,000× *g* for 30 min, diluted 1:2 (v/v) with 50 mM bis-Tris buffer (pH 6.5) and the pH adjusted to pH 6.5. The diluted culture supernatant was applied onto a Q Sepharose column (GE Healthcare Life Sciences, Marlborough, MA, USA) equilibrated in 50 mM bis-Tris (pH 6.5) buffer, and toxins A and B protein peaks were eluted by a NaCl gradient. Toxin A, which was eluted using 200-300 mM of NaCI, was purified further by Chelating Sepharose Fast Flow (GE Healthcare Life Sciences) chromatography as described previously.^12^ Toxin B, which eluted between 500 and 700 mM NaCl, was further purified by chromatography on Mono Q (column size: 8 mL; GE Healthcare Life Sciences), equilibrated in 50 mM bis-Tris (pH 6.5) buffer and eluted with a linear NaCl gradient.

Precursor form of B fragment of binary toxin pCDTb was produced in *Escherichia coli* from a wholly synthetic recombinant gene construct; using an amino acid sequence based on the published sequence from 027 ribotype (http:www.uni-prot.org/uniprot/A8DS70). Detailed methods for cloning, expression and purification of pCDT are detailed elsewhere.^13^

### Antigen microarray

Total specific IgA and IgG binding antibodies against the toxins were determined using a previously validated *C. difficile* protein microarray assay.^14^ Prior to study commencement, we optimized both the dilution of the serum samples and concentration of printed antigens. The purity of all antigens tested in the current study was also evaluated using a silver stain (data not shown). For microarray, stored sera were profiled for total and specific IgA and IgG antibodies to highly purified *C. difficile* whole toxins A (200 μg/mL) and B (100 μg/mL) (toxinotype 0, strain VPI 10463, ribotype 087, obtained from the American Type Culture Collection, Manassas, VA, USA), toxin B from a *C. difficile* toxin-B only expressing strain (CCUG 20309; 90 μg/mL)^15^ and precursor form of B fragment of binary toxin, pCDTb (200 μg/mL). The optimum serum dilution for evaluation of IgA and IgG was 1:100 and 1:500 diluted in an antibody diluent (Dako, Glostrup Municipality, Denmark), respectively. Positive controls incorporated on each plate included tetanus toxoid (National Institute for Biological Standards and Control, Herts, UK) and lysates from *Candida albicans* (Jena Bioscience, Jena, Germany) containing the cytoplasm and cell wall. Negative controls included spotted printed buffer (phosphate-buffered saline; trehalose Tween) and no serum (blank) on each array. Alongside the aforementioned antigens and controls of interest, a human immunoglobulin standard (matching the antibody isotype) consisting of a 10-point two fold serial dilutions from 50 mg/mL to 24.4 ng/mL was spotted onto aminosilane slides (Schott Technical Glass Solutions GmbH, Jena, Germany) in quadruplicates using a MicroGridII arrayer (Digilab, Inc., Marlborough, MA, USA) and a silicon contact pin (Parallel Synthesis Technologies, Santa Clara, CA, USA). Human immunoglobulin standard was used to normalize all the antigens and arrays to a common fluorescence intensity scale.

Slides were scanned at 635 nm and the resultant images were processed with Genepix Pro-6 Microarray Image Analysis software (Molecular Devices Inc., Sunnyvale, CA, USA). Protein signals were determined after background subtraction through customized modules in the *R* statistical language to general mean signal levels. Specific isotype responses were interpolated against the internal isotype standard curve for each sample.

### Toxin neutralization assay

A Caco-2 cell-based neutralization assay for anti-toxin A and anti-toxin B neutralizing antibodies was used to investigate cytopathic effect and cell death as previously published.^16^ Briefly, Caco-2 cells (HTB-37; American Type Culture Collection) were maintained in minimal essential medium plus 20% fetal calf serum, 2 mM glutamine and nonessential amino acids at 37°C. Serum samples were diluted in the assay medium at three dilutions (1:10, 1:100 and 1:1000), then premixed with toxin A or toxin B (at a dose inducing 50% cytopathic effect) for 1 h at 37°C before 50 μL of this mixture was transferred to the cells and incubated for an additional 96 h. Following aspiration of the medium, 50 μL methylene blue (0.5% [wt/vol] dissolved in 50% [vol/vol] ethanol) was added to the cell culture and incubated for 1 h at room temperature. The cells were then washed gently with tap water (to remove excess stain) and air dried. The cells were then lysed by adding 100 μL 1% (vol/vol) N-lauryl-sarcosine and incubated in a shaker for 15 min at room temperature. The cell biomass was determined by measuring the absorbance of each well using a BioTek Synergy2 (BioTeK, Winooski, VT, USA) plate reader at 405 nm. Toxin activity and 50% lethal dose concentrations were defined empirically in preliminary experiments and for each individual batch/lot of toxin used.

### Statistical analysis

All statistical analyses were performed on natural log-transformed data using GraphPad Prism version 6 (GraphPad software, San Diego, CA, USA). The unpaired *t*-test was used to compare the means of two independent samples. For grouped multiple comparisons, the Kruskall–Wallis test and the Dunn’s post hoc test were applied; *p*≤0.05 was deemed statistically significant. Demographic data were presented as medians and ranges.

## Results

The clinical characteristics of the three subject cohorts are highlighted in Table 1. Of the 16 patients in the cystic fibrosis cohort, two and one were found to be carriers of toxigenic and non-toxigenic *C. difficile*, respectively. Compared with patients with *C. difficile* infection, a higher proportion of patients with cystic fibrosis had preexisting diabetes mellitus. However, inflammatory bowel disease was only seen in the *C. difficile*-infected cohort. Although both patient groups had equal exposure to immunosuppressant drugs (defined as corticosteroids, calcineurin inhibitors, mTOR inhibitors, thiopurines and biologics), only patients (n=2) in the cystic fibrosis cohort had previous solid organ transplants. Usage of antibiotics (within the preceding 6 months), proton pump inhibitor/H2 blockers and ursodeoxycholic acid was higher in the cystic fibrosis cohort compared with the *C. difficile*-infected group. In unpaired *t*-tests, there were no significant differences between patients with cystic fibrosis and *C. difficile*-infected cohorts in terms of mean (and standard deviation) white cell counts (8.7 [2.5] vs 9.4 [4.6] × 10^9^/L; *p*=0.59) or lymphocyte counts (1.8 [0.7] vs 1.6 [0.6] × 10^9^/L; *p*=0.35), respectively.

**Figure 1:**
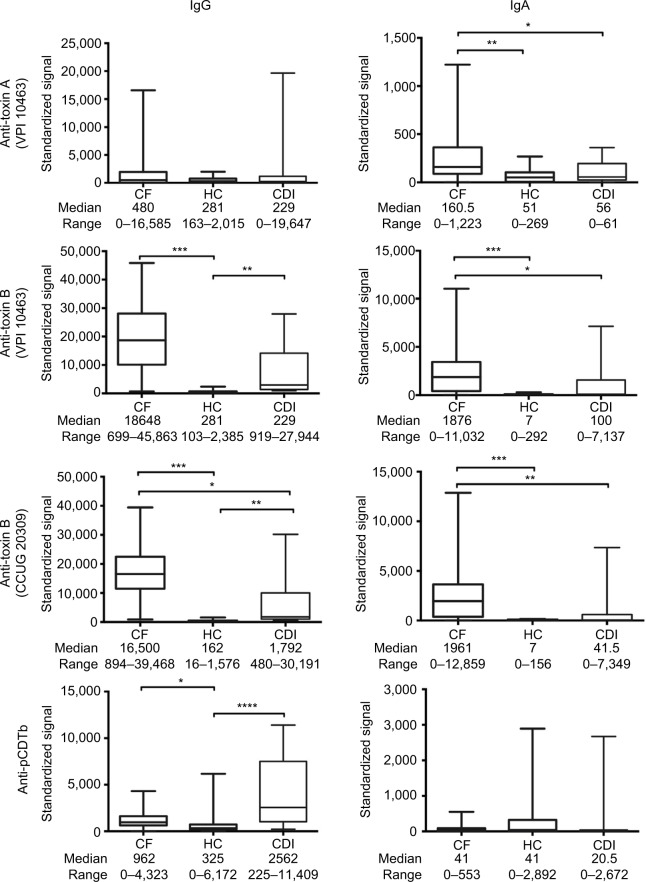
Antibody class-specific responses to *C. difficile* toxins. **Notes:** Serum IgG and IgA antibody responses to *C. difficile* toxins A, B (toxinotype 0, strain VPI 10463, ribotype 087; toxin A at concentration of 200 μg/mL, toxin B at concentration of 100 μg/mL), toxin B (*C. difficile* toxin B-producing strain CCUG 20309; toxin B at concentration of 90 μg/mL) and precursor form of B fragment of binary toxin, pCDTb (200 μg/mL), in patients with CF without diarrhea, CDI with diarrhea and HC. Serum dilution 1:500 for IgG and 1:100 for IgA. Differences between groups were calculated using the Kruskall–Wallis test followed by Dunn’s post hoc test for multiple responses. Box and whisker plots represent the median, range and quartiles. ****p≤0.0001; ***p≤0.001; **p≤0.01; *p≤0.05. Standardized signals are normalized to immunoglobulin standard curve. **Abbreviations:** *C. difficile*, *Clostridium difficile*; CDI, *C. difficile* infection; CF, cystic fibrosis; HC, healthy controls; Ig, immunoglobulin.

Serum samples from patients in the cystic fibrosis group exhibited significantly higher IgA antibody reactivities to *C. difficile* toxins A, B and toxin B (CCUG 20309) but not to pCDTb when compared with the healthy control and *C. difficile*–infected patient groups (Figure 1). Serum IgG antitoxin B (both strains) and anti-pCDTb antibody levels were significantly higher in the cystic fibrosis cohort compared with healthy controls (Figure 1). Systemic IgG anti-toxin B antibody responses predominated and high titer sera did not correlate with high neutralizing potential (data not shown).

**Figure 2:**
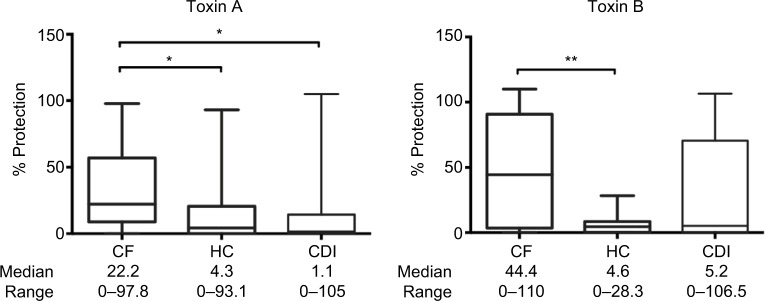
*C. difficile* anti-toxin neutralizing antibody responses in patients’ sera. **Notes:** Neutralizing antibody protection responses against *C. difficile* toxins A and B (toxinotype 0, strain VPI 10463, ribotype 087, used at LD50) in sera (1:100 dilution; toxin A 2.5 ng/mL, toxin B 0.5 ng/mL) from patients with CF without diarrhea, CDI with diarrhea and HC. Differences between groups were calculated using the Kruskall–Wallis test followed by Dunn’s post hoc test for multiple responses. Box and whisker plots represent the median, range and quartiles. **p≤0.01; *p≤0.05. **Abbreviations:** *C. difficile*, *Clostridium difficile*; CDI, *C. difficile* infection; CF, cystic fibrosis; HC, healthy controls; LD50, 50% lethal dose.

Sera in the cystic fibrosis group also contained significantly higher levels of protective neutralizing anti-toxin A and anti-toxin B antibodies compared with the healthy control group (Figure 2).

## Discussion

We identified significantly higher levels of IgA anti-toxins A and B and neutralizing antibodies against toxin A in adult cystic fibrosis patients compared with healthy controls and patients with symptomatic *C. difficile* infection. This same antibody response prevailed for IgG, except that there was no difference in anti-toxin A IgG levels between the groups. Sera from cystic fibrosis patients also exhibited significantly stronger protective anti-toxin neutralizing antibody responses compared with healthy controls (toxins A and B) and patients with *C. difficile* infection (toxin A). Interestingly for pCDTb, IgG levels were also shown to be markedly higher for the cystic fibrosis group compared with the healthy control group. These observations support a previous report that suggests antibody responses to *C. difficile* toxins confer protection against clinical disease.^11^ Although limited by small sample size, our findings suggest that subclinical *C. difficile* infection is common among patients with cystic fibrosis. While both IgG and IgA anti-toxin antibodies may confer protection against symptomatic *C. difficile* infection in the cystic fibrosis population, it is likely that the dominant functional neutralizing antibody effect is exerted by IgG, which is associated with more mature high-affinity responses. When compared to healthy controls and patients with cystic fibrosis, our data are compatible with Islam et al’s study^17^ which showed lower levels of serum anti-toxin IgA in the group with *C. difficile* infection, which might reflect a poor/impaired gut mucosal immune response to toxin-mediated intestinal inflammation. Indeed, in the present study, the IgA binders appeared to be exclusive for toxin A in the cystic fibrosis group whereas antitoxin B associated with both IgG and IgA. While it has been proposed that *C. difficile* toxins may induce paradoxical killing of the fittest B cells and could explain why some individuals are prone to recurrent infections,^18^ we previously detected a higher proportion of circulating toxin-A-specific memory B cells in asymptomatic *C. difficile*-colonized patients with cystic fibrosis compared to non-cystic fibrosis patients with symptomatic *C. difficile* infection.^19^ These findings raise the possibility that patients with cystic fibrosis are able to mount more effective secondary B-cell immune responses that encode protective anti-toxin neutralizing antibodies compared with patients with *C. difficile* infection. Colonization with non-toxigenic *C. difficile* may also protect against colonization with toxigenic strains and may partially explain why patients with cystic fibrosis seldom develop symptomatic *C. difficile* infection.^9^,^20^ Moreover, it is conceivable that differences in colonic mucus or microbiome may protect patients with cystic fibrosis from *C. difficile* infection.

A limitation of this study is the small number of patients and a gender imbalance with a female preponderance in the group with *C. difficile* infection. The latter limitation may reflect the observation that population rates of *C. difficile* infection are commonly reported to be higher in women.^21^ Nevertheless, no gender-related differences in serum antitoxin immunoglobulin levels have been reported in patients with *C. difficile* infection in other studies.^11^,^22^

Recent reports highlight that symptomatic *C. difficile* infection is a significant complication after solid organ and hematopoietic stem cell transplantation in cystic fibrosis and non-cystic fibrosis transplant recipients.^23^ In particular, cystic fibrosis patients who undergo lung transplantation are at a higher risk of developing *C. difficile* infection and seem to present with more atypical/severe disease.^24^ We speculate that a combination of host factors, including both B- and T-cell immunosuppression and/or an altered gut microbiome, is likely to contribute to the elevated risk in this population.

These novel findings, if confirmed in larger studies, may support introduction of seromonitoring to identify transplant recipients displaying low levels of specific anti-toxin antibodies who may be at risk of developing severe *C. difficile* infection. These individuals could be offered prophylactic passive or (when available) active immunotherapies.

## Figures and Tables

**Table 1:** Clinical and demographic characteristics

Subject characteristics	Healthy controls (n=17)	Cystic fibrosis patients (n=16)	Patients with *C. difficile*-associated diarrhea (n=16)
Age (years)	32 (22–89)	28 (19–49)	37 (19–49)
Male/female	8/9	8/8	3/13
Diabetes mellitus	0	11	3
Liver disease	0	3	2
Inflammatory bowel disease	0	0	4
PEG/NG feeding	0	3	1
Previous solid organ transplant	0	2	0
Immunosuppressants	0	5	5
Antibiotic usage (preceding 6 months)	0	16	10
PPI/H2 blocker usage	0	12	4
Ursodeoxycholic acid	0	3	0
Hospitalization (in preceding 6 months)	0	10	9
Previous CDI	0	1	2

**Abbreviations:** CDI, *Clostridium difficile* infection; NG, nasogastric tube; PEG, percutaneous endoscopic gastrostomy; PPI, proton pump inhibitor.
